# Response of the root anatomical structure of *Carex moorcroftii* to habitat drought in the Western Sichuan Plateau of China

**DOI:** 10.1007/s00425-024-04412-3

**Published:** 2024-04-23

**Authors:** Jia-Ying Yang, Hong-Bin Wang, Da-Cai Zhang

**Affiliations:** Key Laboratory of National Forestry and Grassland Administration On Biodiversity Conservation in Southwest China, Southwest Forestry University, Bailongsi 300#, Kunming, Yunnan, 650224 China

**Keywords:** Anatomical structure, Coefficient of variation, Plasticity, Vascular cylinder, Vessel, Soil moisture gradient

## Abstract

**Main conclusion:**

The anatomical structures of *Carex moorcroftii* roots showing stronger plasticity during drought had a lower coefficient of variation in cell size in the same habitats, while those showing weaker plasticity had a higher coefficient of variation. The complementary relationship between these factors comprises the adaptation mechanism of the *C. moorcroftii* root to drought.

**Abstract:**

To explore the effects of habitat drought on root anatomy of hygrophytic plants, this study focused on roots of *C. moorcroftii*. Five sample plots were set up along a soil moisture gradient in the Western Sichuan Plateau to collect experimental materials. Paraffin sectioning was used to obtain root anatomy, and one-way ANOVA, correlation analysis, linear regression analysis, and RDA ranking were applied to analyze the relationship between root anatomy and soil water content. The results showed that the root transverse section area, thickness of epidermal cells, exodermis and Casparian strips, and area of aerenchyma were significantly and positively correlated with soil moisture content (*P* < 0.01). The diameter of the vascular cylinder and the number and total area of vessels were significantly and negatively correlated with the soil moisture content (*P* < 0.01). The plasticity of the anatomical structures was strong for the diameter and area of the vascular cylinder and thickness of the Casparian strip and epidermis, while it was weak for vessel diameter and area. In addition, there was an asymmetrical relationship between the functional adaptation of root anatomical structure in different soil moisture and the variation degree of root anatomical structure in the same soil moisture. Therefore, the roots of *C. moorcroftii* can shorten the water transport distance from the epidermis to the vascular cylinder, increase the area of the vascular cylinder and the number of vessels, and establish a complementary relationship between the functional adaptation of root anatomical structure in different habitats and the variation degree of root anatomical structure in the same habitat to adapt to habitat drought. This study provides a scientific basis for understanding the response of plateau wetland plants to habitat changes and their ecological adaptation strategies. More scientific experimental methods should be adopted to further study the mutual coordination mechanisms of different anatomical structures during root adaptation to habitat drought for hygrophytic plants.

## Introduction

Plant roots directly perceive soil moisture and are the first organ to respond to habitat stress, and the functions of their anatomical structures are enhanced or diminished accordingly (Machado et al. 2013; Chimungu et al. 2015; Donnelly et al. 2016). Plants with different ecological habits exhibit different responses in the face of the same stress (Pregitzer et al. 2002; Galindo et al. 2022). Xerophytes have gradually evolved their own drought-resistant anatomical structures during long-term adaptation to arid habitats (Boughalleb et al. 2014, 2015; Chen et al. 2017) and will change the size of the structures in roots accordingly under drought stress (Brunner et al. 2015), thereby adjusting the strength of their functions in response to stress. Hygrophytes have long been adapted to habitats with high soil moisture content, and root anatomical structures have developed an adaptive relationship with their ecological habits (Singh et al. 2013; Yang et al. 2015; López et al. 2021). For example, in order to adapt to the habitat, the roots of hygrophytes generally have large aerenchyma, which facilitates the transfer of O_2_ from the roots (Xiang et al. 2019). In order to screen the substances entering the roots, hygrophytes also have a strong ectoplasmic barrier structure, so that the root system will not be subjected to the persecution of hazardous substances, which ensures that the plant can be grown normally (Shoaib et al. 2022). Currently, more studies have focused on salinity tolerance and heavy metal uptake in the root anatomy of hygrophytes (Xie et al. 2021), and drought has become an important habitat factor that threatens the survival of hygrophytes. Thus, how will the root anatomy of hygrophytes change when experiencing drought stress?

Root anatomical structures differ in function and also in sensitivity when facing habitat change; that is, their plasticity varies (Karlova et al. 2021). Generally, plasticity is measured according to different plant habitats, and plants with high plasticity have strong adaptability (Yao et al. 2022; Zhang et al. 2022). Some studies have measured the habitat adaptability of plants by the coefficient of variation, and it is believed that if the coefficient of variation of plant morphology is large in different habitats, that is, if the plant morphology is diversified, the plant habitat adaptability is strong (Liu et al. 2018; Pang et al. 2019). It was shown that the plasticity of the anatomical structure and function of the root and the coefficient of variation of the morphology are particularly important for the plant to face the habitat changes, which is directly related to the physiological function of the root system, and thus affects the habitat adaptability of the plant (Zhu et al. 2015; Liu et al. 2016; Cris et al. 2019). In both cases, the adaptive capacity of structural functions and the coefficient of variation are measured as a single indicator, whereas in root anatomy, the horizontal transport structure (including the epidermis and exodermis) mainly absorb water and nutrients and transport them to the vascular cylinder, and the vertical transport structure (including the vascular cylinder and vessels) transport water and nutrients to the other organs of the plant (Eissenstat et al. 1999; Geng et al. 2018; Du et al. 2018; Kim et al. 2020). In the process of root adaptation to the environment, root anatomical structures may increase functional plasticity by increasing the functionality of horizontal and vertical transport structures, or may increase the degree of variability by increasing trait differences between horizontal and vertical transport structures to resisting habitat changes. Currently, the functional adaptation and coefficient of variation of plant anatomical structures were measured in different habitats, and few studies have linked the functional adaptation of plant anatomical structures in different habitats to the variation degree of plant anatomical structures in the same habitat. Whether the relationship between these two indicators reveals new mechanisms of plant adaptation to different habitats is also unknown.

The wetlands on the Western Sichuan Plateau of China have a large area and are rich in hygrophytes, but under the dual interference of global climate change and human disturbance, the area of wetlands is rapidly decreasing (Xu et al. 2019; Zhang et al. 2021), and the living space for hygrophytes is diminishing. As a dominant species in the wetlands of the western Sichuan Plateau, *Carex moorcroftii* has the function of soil and water conservation and maintaining ecological balance; with the drought of the habitat, the importance value of *C. moorcroftii* in the plant community gradually declined (Xiao et al. 2014), and a lot of researches have been carried out on its geographic distribution, morphological characteristics and biomass in this process (Liu et al. 2018; Wang et al. 2021). During drought, the root anatomy of *C. moorcroftii* exhibit strong adaptations in functional traits, but the response mechanism is unclear.

To explore the effects of habitat drought on root anatomy of hygrophytic plants, our research focuses on the dominant species in the wetlands of the Western Sichuan Plateau.* C. moorcroftii* samples were collected along a gradient of habitat drought to analyze the relationship between root anatomical structure and ecological habits, variations in root anatomical structure characteristics along soil moisture gradients, the sensitivity of different anatomical structures to changes in soil moisture, and the ability of roots to adapt to habitat changes.

## Materials and methods

### Study site

**Fig. 1:**
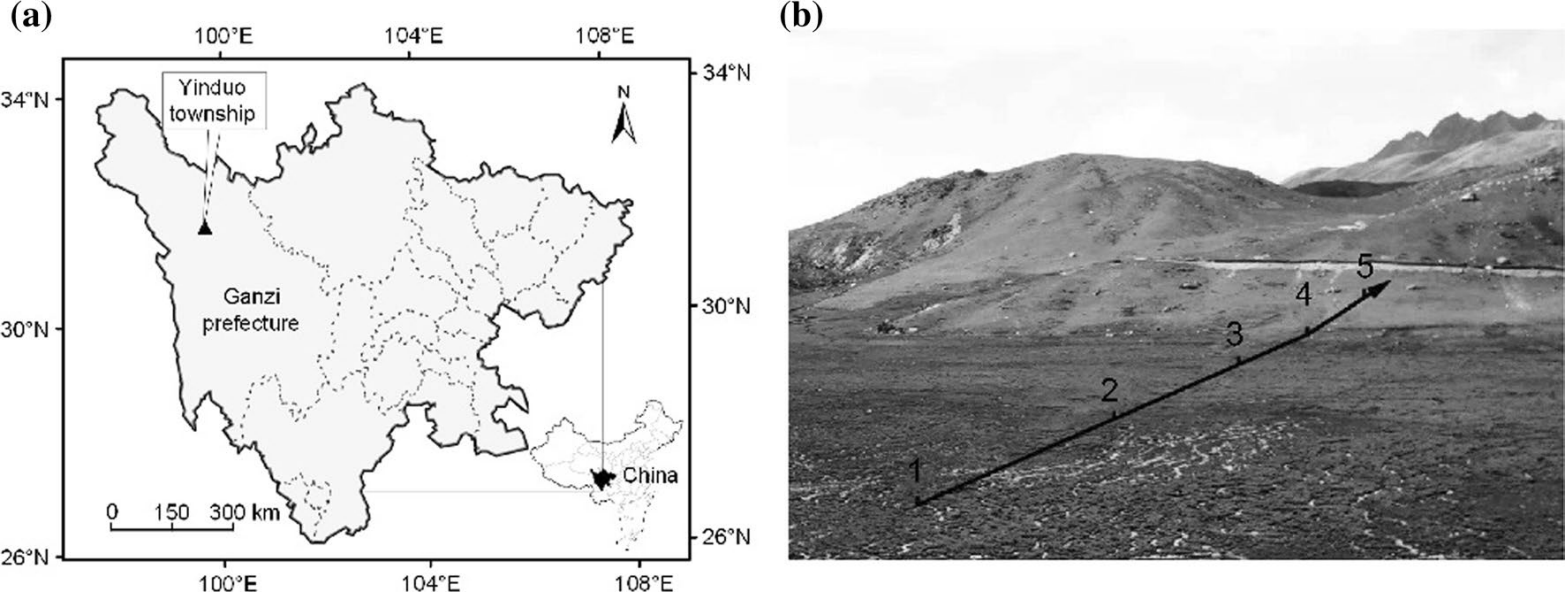
Map of research setting (**a**) and sample plot setting (**b**)

The study site was located in the ancient ice cap wetland in Yinduo Township, Xinlong County, Ganzi Prefecture, Sichuan Province, China, with geographic coordinates 31°24′25″ N, 99°49′48″ E and elevation of 4300–4450 m (Fig. 1a). It belongs to the subhumid climate zone of the Qinghai-Tibet Plateau. The terrain is plateau and mountainous, and the area between the two mountains is gentle and has a large area of swampy waters. *C. moorcroftii* is the monodominant species in swampy meadows in shallow-water areas. Alpine meadows are located on slopes, and the codominant species include *Kobresia humilis*, *K. macrantha*, and *C. moorcroftii*, and the accompanying species include *C. atrofusca* var. *minor*, *Poa alpina*, *P. arctica*, and *Stipa capillacea*. The study site formed a natural arid gradient from aquatic to humid habitats, which could objectively reveal the changes of anatomy with the drought of habitats for *C. moorcroftii*.

### Material collection

Five 4 m × 4 m sample plots were set up from the low-lying flowing water area to the alpine meadow, among which two plots were located in the marsh meadow and three plots were located in the *Kobresia* meadow (Fig. 1b). *C. moorcroftii* were distributed in the five sample plots but with different spatial distribution patterns (Table 1). The image method (Guan et al. 2010) was used to measure the cover of *C. moorcroftii* in each sample plot, and a TSC-IW soil moisture meter was used to measure the volume moisture content of soil. The materials were collected in August 2018, and more than 30 plants of *C. moorcroftii* were randomly collected from each plot. One new white root was chosen from each plant, cleaned and placed in FAA fixative (HCHO:CH_3_COOH:70% C_2_H_5_OH = 1:1:18, by vol.).

**Table 1:** Information on the sample plots

Sample plot no	Terrain and habitat	Soil moisture content (%)	Spatial distribution pattern	C. moorcroftii cover (%)
1	Plain, swamp meadow	100	Uniformed	95
2	Plain, swamp meadow	80.07	Clustered	60
3	Gentle slope, alpine meadow	68.39	Random	25
4	Gentle slope, alpine meadow	51.04	Random	15
5	Steep slope, alpine meadow	28.78	Random	5

### Paraffin method

The root was taken out of the fixative and an approximately 1-cm segment of the root was cut in half at 0.5 cm from the root tip. The samples were first washed with 70% ethanol and dehydrated with an increasing concentration series (70%, 85%, 90% and 100%) of tert-butanol-ethanol solutions for 2 h. Then, the samples were cleared with 100% tert-butanol 3 times for 2 h each time. Once transparent, the material was immediately dipped in pure paraffin wax and placed in a constant-temperature oven at 63 °C for 2 to 3 days. Then, the samples were embedded with a KD-BM II tissue embedding machine, sliced with a Leica RM 2145 microtome at a thickness of 8 to 10 μm, dried in a 40 ℃ oven, and dyed with safranin O–fast green (Cortaga and Sebidos 2019). Fifteen roots were taken from each plot, and each root has 2 slices. Thus, a total of 150 slices were taken from the 5 plots.

### Data acquisition

The slices were viewed under a Leica DM 500 optical microscope, and images were obtained using LAS V4.4 software. More than 600 photos were obtained. ImageJ software was used to measure root transverse diameter (μm) and area (A_1_, μm^2^), epidermal cell thickness (μm), exodermis layer number (N_1_) and thickness (μm), aerenchyma area (A_2,_ μm^2^), Casparian strip thickness (μm), vascular cylinder diameter (μm) and area (A_3_, μm^2^), and vessel number (N_2_), vessel area (A_4_, μm^2^) and vessel diameter (μm). Here, the thickness of the Casparian strip, exodermis and epidermal cells and the average area and average diameter of vessels were expressed as the mean of ten continuously distributed cells; the diameters of the vascular cylinder, root cross-section, and vessels were expressed as the mean of the horizontal and vertical diameters. According to the measurement data calculation, the vascular cylinder area ratio = ${A}_{3}/{A}_{1}$, aerenchyma area ratio = ${A}_{2}/{A}_{1}$, total vessel area = ${A}_{4}\times {N}_{2}$, and total vessel area ratio = $\left({A}_{4}\times {N}_{2}\right)/{A}_{3}$.

### Statistical analysis

Microsoft Excel software was used for data recording and sorting, and SPSS 26.0 software was used for data analysis. One-way ANOVA was used to analyze the difference in root anatomical structures among soil moisture levels (*P* < 0.01), and linear regression was used to analyze the variation in root anatomical structures along a soil moisture gradient. Canoco 5.0 software was used to analyze the ranking relationship between root anatomical structures and soil moisture content, and the ranking model was selected based on the gradient length in the detrended correspondence analysis results (Morris et al. 2015). In this study, when the maximum gradient length was greater than 3, the redundancy analysis linear ranking model was chosen. The first axis of the sorting diagram was taken as the horizontal axis (− 1,1) and divided into three sections as the basis for the classification of plasticity strength. The first section is (− 0.25, 0.25), and the plasticity is weak. The second section is (− 0.5, − 0.25) and (0.25, 0.5), and the plasticity is medium. The third section is (− 1, − 0.5) and (0.5,1), and the plasticity is strong. The first axis of the sorting diagram was taken as the horizontal axis (− 1, 1) as the basis for plasticity strength, and the length of the arrows projected to the first axis of each anatomical structure was divided by the sum of the projected lengths of each index to measure the functional adaptation of the root anatomical structures in different soil moistures and was denoted plasticity 1. Coefficients of variation = $\left(\frac{SD}{M}\right)100\%$. The difference between the maximum and minimum coefficients of variation of each anatomical structure under different soil moisture contents divided by the total difference was used to measure the variation degree of the root anatomical structures at the same soil moisture level and was denoted plasticity 2. Data were plotted with Origin 2018 software.

## Results

### Anatomical characteristics of roots and variations in root transverse section area along a soil moisture gradient

**Fig. 2:**
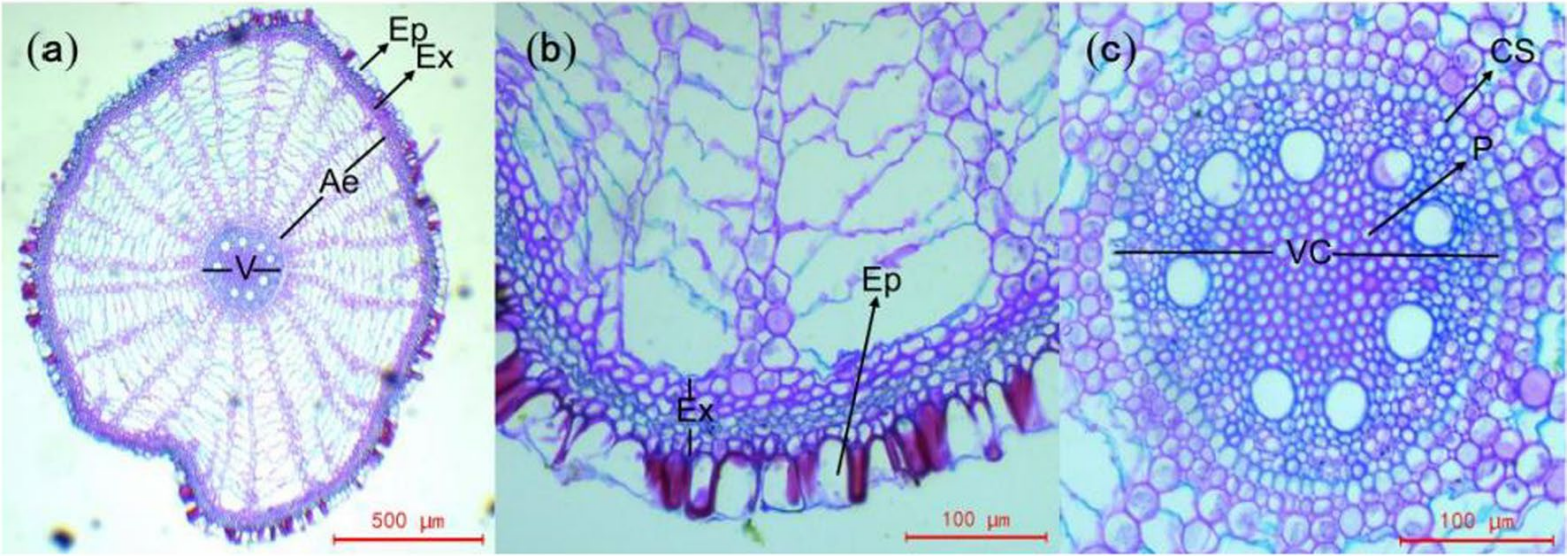
Anatomical structure of the *C. moorcroftii* root. **a** Root transverse section. **b**, **c** Part of the root transverse section at higher magnification. Ep: Epidermis; Ex: Exodermis; Ae: Aerenchyma; CS: Casparian strip; VC: Vascular cylinder; P: Pith

The transverse section of the root is an irregular circle (Fig. 2a). The epidermal cells are an oblong monolayer with a stratum corneum. The exodermal cells are tightly packed, with 3 to 5 layers (Fig. 2b). Lysigenous aerenchyma accounts for 51–71% of the root transverse section area (Fig. 2a). The cells of the endodermis are arranged in a regular circle and thickened by "U" embolization to form the Casparian strip. The vascular cylinder is nearly round and consists of primary xylem, primary phloem, and parenchyma, with 7 to 10 rows of vessels (Fig. 2c). The pith is clearly visible, and the cell wall is thickened (Fig. 2c).

**Fig. 3:**
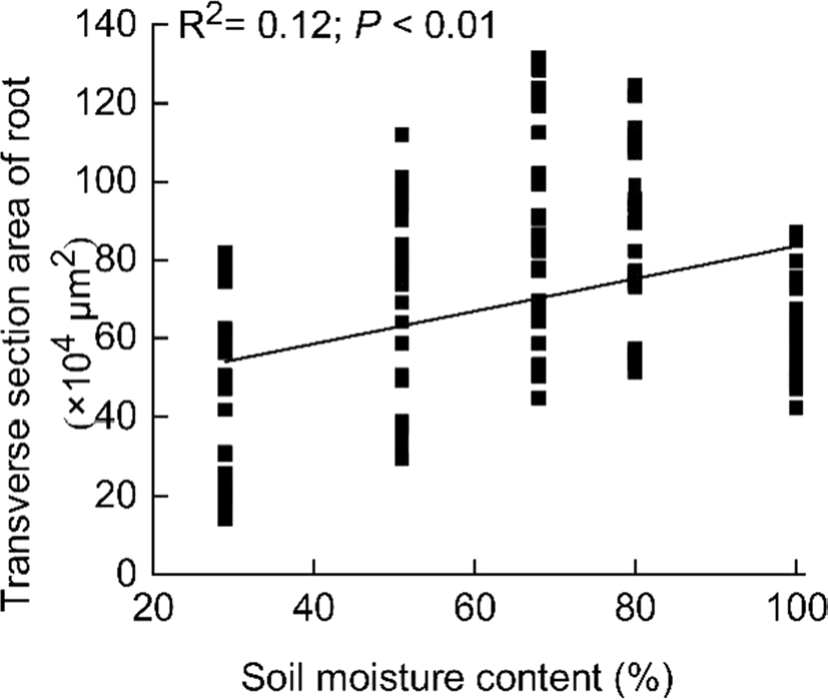
Variations in root transverse section area correlated with soil moisture gradient. Mean values ± SE, *n* = 150

The area of the transverse section ranged from 37.4 to 92.5 × 10^4^ μm^2^, and is significantly and positively correlated with the soil moisture gradient (*P* < 0.01; Fig. 3).

Variations in the horizontal transport structure of roots along the soil moisture gradient.

**Fig. 4:**
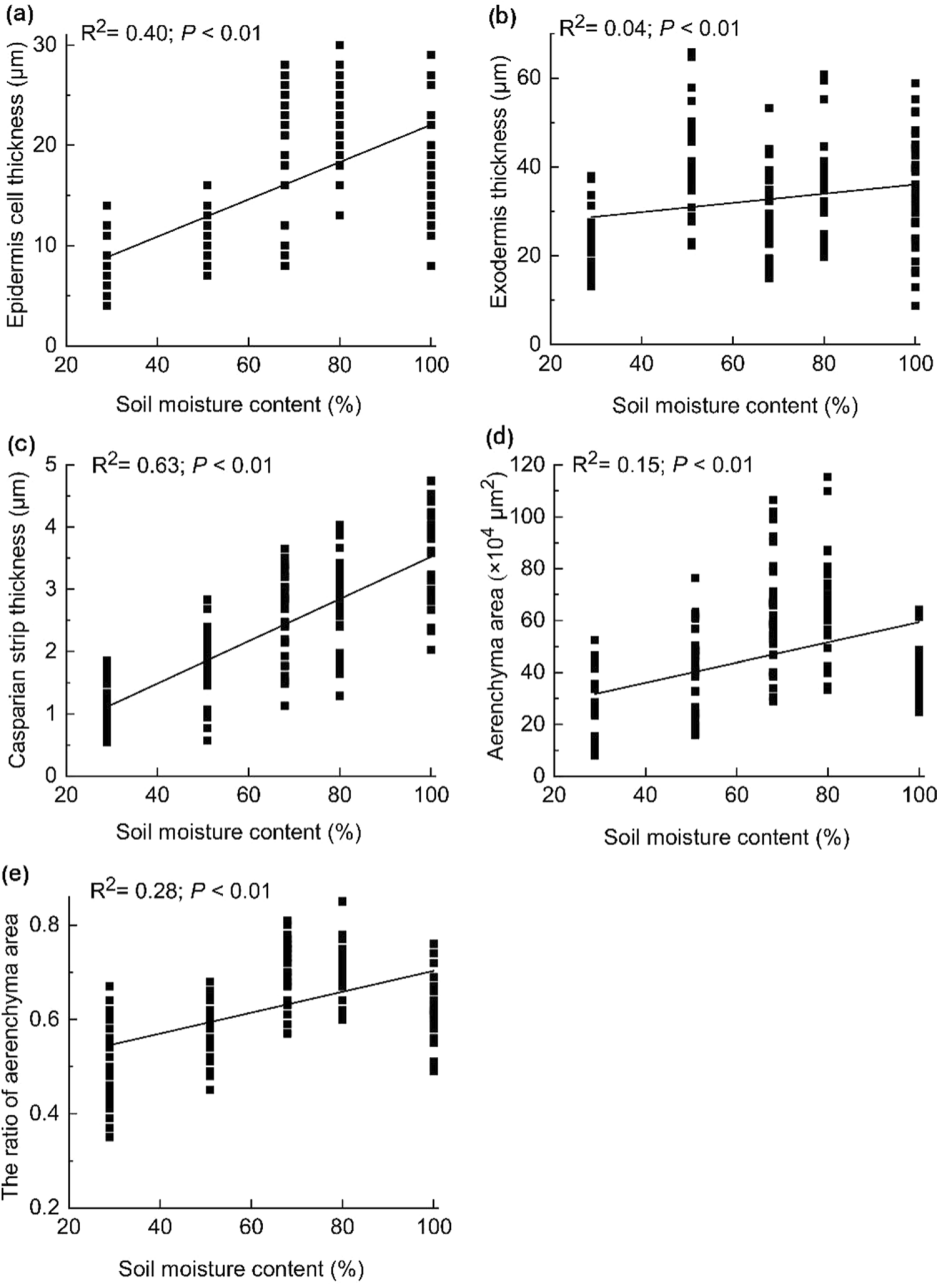
Variations in the horizontal transport structure in relation to the soil moisture gradient. Mean values ± SE, *n* = 150

The epidermal cells of the root ranged in thickness from 7.93 to 22.63 μm (Fig. 4a), and those of the exodermis from 23.08 to 40.80 μm (Fig. 4b). The thickness of the Casparian strip ranged from 1.16 to 3.55 μm (Fig. 4c), the aerenchyma area ranged from 19.93 to 66.51 × 10^4^ μm^2^ (Fig. 4d), and the ratio of the aerenchyma area to the root transverse section area ranged from 0.51 to 0.71 (Fig. 4e); all of which were significantly and positively correlated with the soil moisture gradient (*P* < 0.01; Fig. 4).

### Variations in the vertical transport structure of roots along the soil moisture gradient

**Fig. 5:**
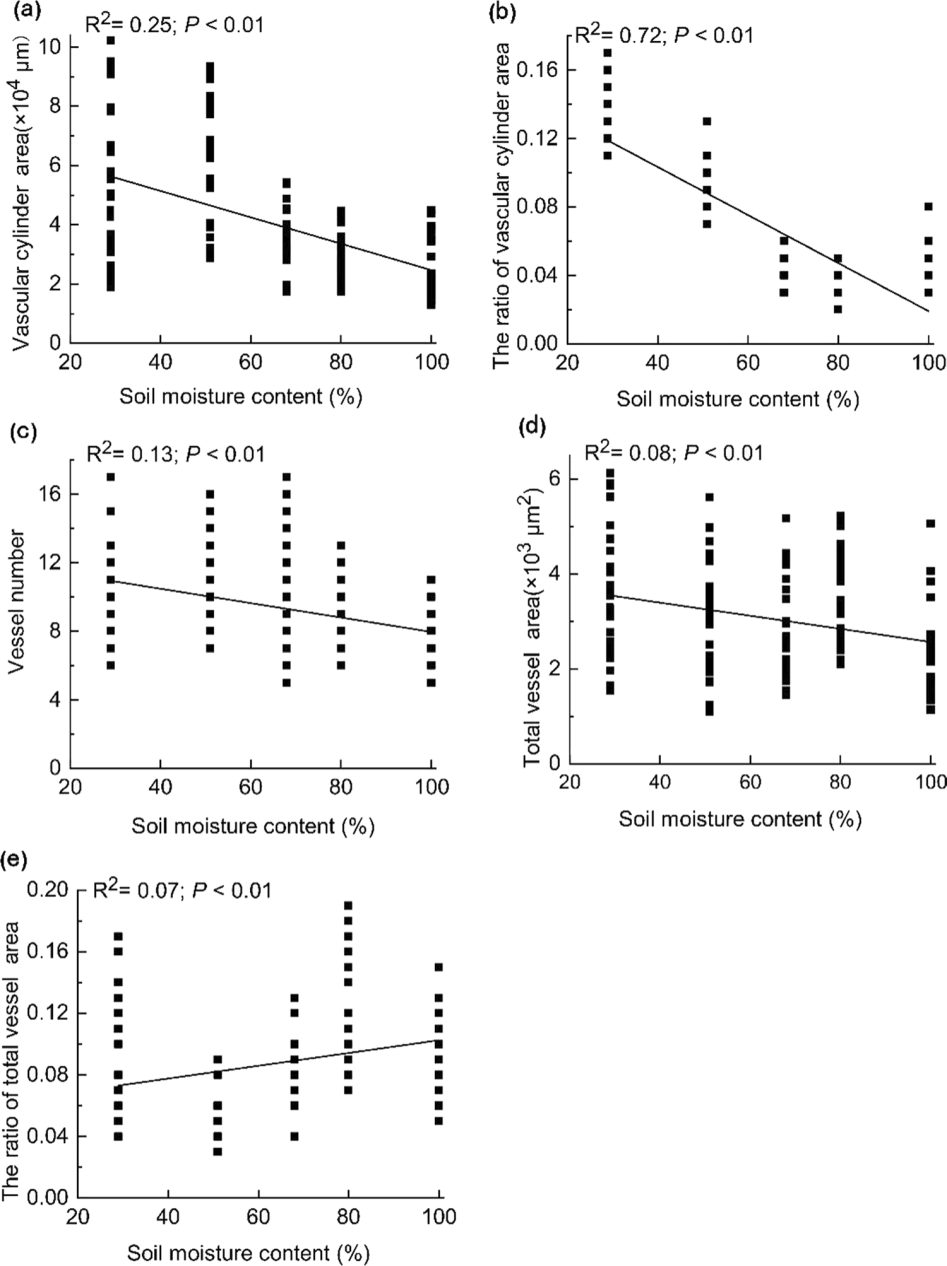
Variations in the vertical transport structure in relation to the soil moisture gradient. Mean values ± SE, *n* = 150

The area of the vascular cylinder was 2.49 to 6.40 × 10^4^ μm^2^ (Fig. 5a), the ratio of vascular cylinder area to transverse section area was 0.03 to 0.13 (Fig. 5b), and the number of primary xylem vessels was 7 to 11 rows (Fig. 5c). The total area of vessels ranged from 2.31 to 3.63 × 10^3^ μm^2^ (Fig. 5d), and all were significantly and negatively correlated with the soil moisture gradient (*P* < 0.01; Fig. 5a–d). The ratio of total vessel area to vascular cylinder area ranged from 0.05 to 0.12, and was significantly and positively correlated with the soil moisture gradient (*P* < 0.01; Fig. 5e).

### Plasticity analysis of root anatomical structures

**Fig. 6:**
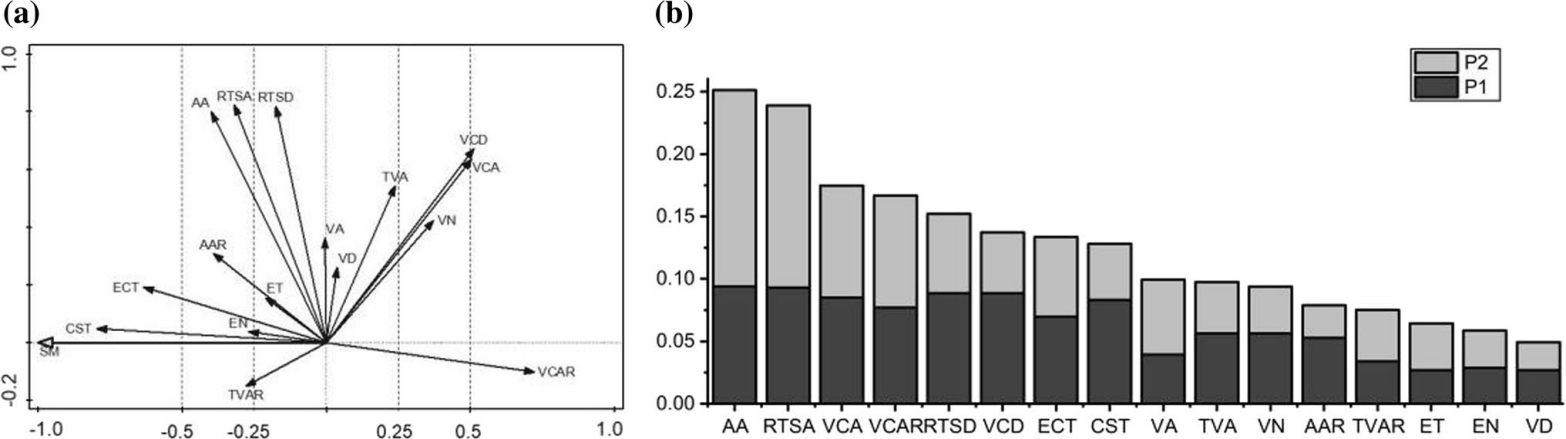
Redundancy analysis ordination diagram of root anatomical structures and soil moisture gradient (**a**) and plasticity 1 and plasticity 2 of each anatomical structure (**b**). *SM* soil moisture, *P1* plasticity 1, *P2* plasticity 2, *VCAR* vascular cylinder area ratio, *CST* Casparian strip thickness, *ECT* epidermis cell thickness, *VCD* vascular cylinder diameter, *VCA* vascular cylinder area, *AA* aerenchyma area, *AAR* aerenchyma area ratio, *RTSA* root transverse section area, *VN* vessel number, *TVAR* total vessel area ratio, *EN* exodermis layer number, *TVA* total vessel area, *ET* exodermis thickness, *RTSD* root transverse section diameter, *VD* vessel diameter, *VA* vessel area. Values (− 0.25, 0.25), the plasticity is weak; − 0.5, − 0.25) and (0.25, 0.5), the plasticity is medium; (− 1, − 0.5) and (0.5, 1), the plasticity is strong

The plasticity of the *C. moorcroftii* root anatomical structures can be divided into three types: strong plasticity, medium plasticity and weak plasticity. The structures with strong plasticity included the ratio of the vascular cylinder area to root transverse section area, thickness of the Casparian strip, thickness of epidermal cells, and diameter and area of the vascular cylinder. The medium plasticity structures were composed of the aerenchyma area and area ratio, root transverse section area, number of vessels, ratio of total vessel area to root transverse section area, and number of exodermal layers. The weak plastic structures included the total vessel area, epidermal thickness, diameter of root transverse section, and diameter and area of vessels (Fig. 6a).

In the driest habitat, the coefficient of variation was the smallest for the vascular cylinder area ratio, which thus showed the strongest functional adaptation, and was the largest for the total vessel area, which thus showed the weakest functional adaptation (Table 2). In addition, the vascular cylinder area ratio, total vessel area, exodermis thickness and vessel diameter plasticity 1 and plasticity 2 were symmetrically distributed, while the plasticity 1 and plasticity 2 of the remaining anatomical structures were asymmetrically distributed (Fig. 6b).

**Table 2:** Coefficient of variation of root anatomical structures in different soil moistures (CV%)

SM	VCAR	CST	ECT	VCD	VCA	AA	AAR	RTSA	VN	TVAR	EN	TVA	ET	RTSD	VD	VA
100	0.34	0.21	0.27	0.18	0.38	0.27	0.12	0.20	0.20	0.38	0.26	0.38	0.38	0.32	0.17	0.28
80	0.20	0.27	0.17	0.15	0.29	0.30	0.09	0.24	0.21	0.28	0.18	0.28	0.33	0.15	0.14	0.29
68	0.18	0.27	0.38	0.15	0.29	0.34	0.09	0.31	0.30	0.36	0.20	0.36	0.30	0.15	0.22	0.42
51	0.17	0.30	0.21	0.17	0.33	0.40	0.10	0.34	0.25	0.39	0.18	0.39	0.28	0.19	0.19	0.28
29	0.10	0.33	0.34	0.28	0.53	0.69	0.16	0.59	0.28	0.35	0.22	0.35	0.31	0.30	0.15	0.26

## Discussion

### Relationship between root anatomical structures and ecological habit

The anatomical structures of the roots of *C. moorcroftii* were similar to those of most monocotyledonous plants in wetlands (Soukup et al. 2005; Yang et al. 2019a), but there are differences in the ratio of the structures. First, the ratio of aerenchyma area to the transverse section area of the root is 0.51–0.71 (Fig. 2a), which is extremely high. Aerenchyma is an oxygen channel for plants to survive in aqueous habitats (Efremov et al. 2016), and a larger area ratio indicates a higher water content in the plant habitat (Leandro et al. 2022). This ratio is still high in the driest habitats, indicating that the ecological habits of *C. moorcroftii* are not easily changeable. Second, the ectoplasmic barrier structure is prominent (Fig. 2b, c), including the exodermis, endodermis and Casparian strip, which is consistent with the anatomical structure of the root of *Eichhornia crassipes* (Madhubala et al. 2021). The nutrition and water of roots in wet habitats need to be controlled by barrier structures (Andersen et al. 2021; Beisson et al. 2012) to prevent harmful substances from entering the root vascular cylinder and prevent the loss of nutrients from the roots, which are the fundamental structures of plant hygrophytes (Barberon et al. 2016). These structures, formed in the process of long-term habitat evolution, have given rise to the ecological habits of *C. moorcroftii* and have ensured its dominance in wetlands.

### Adaptation of root anatomical structures to soil moisture gradient

The response strategies of the horizontal and vertical transport structures of the *C. moorcroftii* root to drought are different. During drought, the transverse section area of roots decreases with decreasing soil moisture (Fig. 3), and the horizontal and vertical transport structures exhibit opposite changing trends. The thickness of the epidermis and exodermis, the area of the aerenchyma, the ratio of the aerenchyma area to the root transverse section area, and the thickness of the Casparian strip were all significantly and positively correlated with soil moisture content (Fig. 4). The epidermis absorbs water, but epidermal cells also consume water through respiration in water-deficient habitats (Xiao et al. 2020; Halder et al. 2022; Kou et al. 2022). Therefore, reducing the thickness of the epidermis is a water retention strategy in *C. moorcroftii*. When water enters the root vascular cylinder, it is first transported horizontally, and the thickness of the exodermis is related to the horizontal water transport efficiency, so reducing the thickness of the exodermis is a manifestation of reduction in the horizontal water transport distance in *C. moorcroftii*. Aerenchyma is a channel for oxygen in wet and hypoxic habitats (Bartlett et al. 2022), so it has less significance in arid habitats, resulting in a decrease in its area and in the ratio of its area to the root transverse section area. The change in the thickness of the Casparian strip is contrary to the research results of increased Casparian strip thickness when *Populus przewalskii* responds to arid habitats (Yu et al. 2020), because *C. moorcroftii* is a hygrophytic plant, and the Casparian strip is more adapted to wet habitats. As one of the structures of the extracellular barrier, the Casparian strip mainly has the function of preventing harmful substances from entering the root vascular cylinder in wet habitats. However, in arid habitats, Casparian strips have less significance, which means that reducing their thickness is beneficial for water transport. Compared with the ability of xerophytes to absorb and retain water in response to drought by increasing the thickness of the epidermis and Casparian strip (Zhao et al. 2011; Grzesiak et al. 2019), *C. moorcroftii* has its own special drought adaptation mechanism, that is, to reduce the thickness of the epidermis and Casparian strip and the area of each horizontal transport structure to increase the efficiency of water horizontal transport.

The area of the vascular cylinder, ratio of vascular cylinder area to root transverse section area, number of vessels, and total area of vessels were significantly and negatively correlated with soil moisture (Fig. 5). The vascular cylinder transports water and nutrients vertically in plants (Gu et al. 2014) and is closely related to plant mechanical support (Liu et al. 2005). The number and area of vessels are directly related to the efficiency of water transport (Köcher et al. 2012; Wang et al. 2022). The efficiency of water transport in arid habitats is particularly important; therefore, by increasing the total area of vessels, *C. moorcroftii* increases the ratio of the area of the vascular cylinder to that of the root transverse section area and thus increases the water transport efficiency. The ratio of vessel area to vascular cylinder area decreases with decreasing soil moisture, indicating that other structures of the vascular cylinder also increase in size to increase the ratio of the vascular cylinder to the root, enhancing the efficiency of vertical water and nutrient transport as well as their own mechanical support.

### Ability of root anatomical structures to adapt to habitat

The anatomical structures of the roots of *C. moorcroftii* have strong adaptability to different habitats. Except for the weak plasticity of the diameter and area of the vessels, thickness of the epidermis, and diameter of the root transverse section, all other structures have strong plasticity, including the vascular cylinder diameter, area and area ratio (Fig. 6a). Plasticity reflects the sensitivity of root anatomical structures to soil moisture (Hou et al. 2017; Potocka et al. 2018; Shelden et al. 2023). When soil moisture is lacking, the vascular cylinder in the *C. moorcroftii* root quickly responds to drought and increases its area in arid habitats. As the main water transport structure in the vascular cylinder, the number of vessels gradually increases with decreasing soil moisture, and the plasticity of the number of vessels is stronger than that of vessel diameter, which indicates that the number of vessels is the first parameter to increase in response to arid habitats. This is similar to the strategy of *Bombax ceiba* to improve water delivery efficiency, in which the diameter of the vessels is reduced and the number of vessels is increased in dry and hot valleys (Zhao et al. 2016). In the absence of soil moisture, longer vessels and narrower lumens will not cause lumen embolism (Ding et al. 2016; Wang et al. 2019; Li et al. 2022), which is more conducive to vertical water transport over long distances.

Moreover, the vascular cylinder area ratio, the total vessel area, exodermis thickness and vessel diameter plasticity 1 and plasticity 2 are symmetrically distributed, while the plasticity 1 and plasticity 2 of the Casparian strip thickness, epidermis cell thickness, aerenchyma area, root transverse section area, vessel number, exodermis number and exodermis thickness are asymmetrically distributed and plasticity 1 and plasticity 2 form a complementary relationship (Fig. 6b). When the functional adaptation of plant anatomical structures is strong in different habitats, the variation coefficient of plant anatomical structures is low in the same habitats, indicating that the plant anatomical structures were more sensitive to habitat changes (Schneider et al. 2022). Similarly, Casparian strip thickness, exodermis number, epidermis cell thickness and vessel number in the root anatomical structure of *C. moorcroftii* showed strong structural functional plasticity with low coefficients of variation in the soil moisture gradient, indicating that these structures have a major role in enhancing structural function in response to habitat drought. When the functional adaptation of plant anatomical structures is weak in different habitats, the variation coefficient of plant anatomical structure is high in the same habitats, indicating that the plant shows structural diversity in changing habitats (Yang et al. 2019b; Zhao et al. 2016). *C. moorcroftii* show strong variability in the exodermis thickness, aerenchyma area, and root transverse section area, while structural functional plasticity is low, indicating that these structures have a major role in enhancing structural diversity in response to habitat drought. At the population level, nutrients and water of *C. moorcroftii* can be transported among clones through the asexual network formed by the clonal building constructs, enabling resource sharing across the asexual lineage as a means of improving the population's adaptive capacity to the habitat (Liu et al. 2009). In our study, at the individual plant level, the simultaneous changes in the functional adaptation of root anatomical structures in different habitats and the variation degree of root anatomical structures in the same habitats, and this complementary relationship between structural function and structural diversity make the habitat adaptability of *C. moorcroftii* stronger.

## Conclusion

The aerenchyma, which accounts for more than half of the area of the root transverse section and is the extracellular barrier structure controlling the entry of harmful substances into the root vascular cylinder of *C. moorcroftii*, is formed by long-term adaptation to wetland habitats and has achieved its own ecological habits. During drought, *C. moorcroftii* adopts the strategy of shortening the horizontal water transport distance while increasing the number of vessels to accelerate vertical water transport to enhance its ability to resist drought. At the same time, the functional adaptation of root anatomical structures in different habitats and the variation degree of root anatomical structures in the same habitats are complementary, and the two factors coordinate with each other to form a unique adaptation mechanism, which makes *C. moorcroftii* more adaptive to changes in its habitat. The results of this study further supplement the research on the response of plants to habitat drought in plateau wetlands, and provide a scientific basis for the response of plateau wetlands to habitat drought. Based on the current context of habitat drought, it is necessary to increase the depth and breadth of research on more plateau wetland plants in the future, and to observe the changes of wetland plants from all nutrient organs of plants, in order to have a more comprehensive understanding of wetland plants response to habitat changes.

## Data Availability

The data sets generated and/or analysed during the current study are available from the corresponding author on reasonable request.
